# To manage long COVID by selective SARS-CoV-2 infection biosensing

**DOI:** 10.1016/j.xinn.2022.100303

**Published:** 2022-08-11

**Authors:** Ajeet Kaushik, Ebrahim Mostafavi

**Affiliations:** 1 NanoBiotech Laboratory, Department of Environmental Engineering, Florida Polytechnic University, Lakeland, FL, USA; 2 Stanford Cardiovascular Institute, Stanford University School of Medicine, Stanford, CA 94305, USA; 3 Department of Medicine, Stanford University School of Medicine, Stanford, CA 94305, USA

**Keywords:** long COVID, SARS-CoV-2 infection, biosensor, point-of-care systems, personalized COVID-19 management

According to the National Institutes of Health (NIH), the initiative of The Rapid Acceleration of Diagnostics Tech and Advanced Technology Platforms (RAD_x_Tech/ATP) program was established to promote research and development (R&D) in the field of investigating selective, sensitive, and affordable sensing technologies for coronavirus disease 2019 (COVID-19) diagnostics. This is a goal-oriented approach toward point-of-care testing (POCT) and severe acute respiratory syndrome coronavirus 2 (SARS-CoV-2) infection management applications. Till May 2022, RAD_x_Tech/ATP has been involved in 1.9 billion tests production, 44 FDA authorized tests, first over-the-counter test for POCT and home-based testing, and >100 companies dedicated to smart sensor development (https://www.nibib.nih.gov/covid-19/radx-tech-program/radx-tech-dashboard). Despite such outstanding growth, COVID-19 management is far from in control due to SARS-CoV-2 mutations, virus presence in life systems (including human-to-human transmission, water, food, animal, and air [mainly indoor premises]), and long-term post-infection consequences especially in a situation where re-infection appears as an emerging challenge. It is reported that 2.5% of COVID-19 patients exhibited serious post-covid symptoms. This is a huge number and may often need SARS-CoV-2 testing to track the possibilities of re-infection and risk assessments. In some cases, the organ damage (lung tissue) and severe inflammation due to pathogenesis of SARS-CoV-2 have appeared as permanent or long-lasting problems. Therefore, developing smart sensing systems (SSSs) suitable for POCT will be a key factor to manage long COVID.^1^, ^2^, ^3^ In such scenarios, i.e., long COVID, which is also the focus of this editorial, the role of SSSs becomes more essential for the selective detection of mutated SARS-CoV-2 at very low concentrations, in a reduced form factors manner, to manage long COVID intelligently.

## State-of-the-art SARS-CoV-2 biosensing

The two most reliable methods to detect and monitor COVID-19 (RT-PCR and computerized tomography [CT] scan) were limited to adaptability for large populations because of their multiple technical requirements. The COVID-19 test has already been greatly scaled up today by optimizing the test procedure (for example, COVID-19 diagnostic tests by PT-PCR were commonly proceeded by adopting a 20 mixed 1 sample strategy [even 50 mixed 1 sample in some cases] in China). So, the scalability of COVID-19 using the RT-PCR test was unlikely the main reason for the requirement to develop other POCTs for COVID-19 pandemic control at its current state, but it was the high transmission risk of SARS-CoV-2 considering the crowd-gathering demand when tested, the strong infectiousness of SARS-CoV-2, and its presence in the life cycle.

To achieve testing at a large scale, lateral flow assay (LFA)-based qualitative diagnostics of COVID-19 were very useful to perform laboratory testing and POCT due to the easy scaling-up capability. However, LFA-based COVID-19 diagnostics exhibited the issue of false-positives, so they are not recommended for detecting SARS-CoV-2 at low levels. Presently, there are dozens of LFA-based COVID-19 diagnostic kits on the market, which mainly focus on the detection of nucleocapsid or spike (S-1) protein antigen of SARS-CoV-2. Such LFA methods are especially suitable for self-testing for their extremely simple operation and read-out, which is probably the most outstanding advantage over RT-PCR and CT scan in the lab. Such self-testing diagnostics can minimize the transmission risk of SARS-CoV-2 by avoiding crowd gathering. Yet those kits could only serve as a preliminary screening stage for large populations because of their inferior technical performance including detection limit, false-positive, and false-negative interferences, etc. So, there are merits of such LFA-based COVID-19 diagnostic kits include scaling-up capability indeed, yet the isolation advantage in diagnosing contagious SARS-Cov-2 should play the key role. For LFA-based COVID-19 diagnostic kits, their poor performance at detecting SARS-CoV-2 at a low level should lead to false-negative results remarkably, but not to false-positive results. The detection specificity issue could lead to both false-positive (other virus interference) and false-negative results (likely due to mutation).

The need for COVID-19 management was always the primary recommendation due to several reasons including but not limited to (1) unavailability of effective treatments to combat SARS-CoV-2 infection, (2) easy human-to-human transmission of the virus, (3) SARS-CoV-2 causes severe respiratory diseases along with damaging other organs, (4) presence of SARS-CoV-2 in the life cycle including water (outlets of buildings and households), animals (cats, deer, etc.), food (meat, dairy products, food, etc.), and air (indoor air, hospital-acquired infections, buildings, etc.), (5) inevitable and frequent mutations in the SARS-CoV-2 variants of concern such as Alpha, Beta, and Gamma (first wave, April 2020), Delta (second wave, July 2020), and Omicron (third wave, Nov 2021), 6) to monitor the efficacy of the treatment where the viral load variation was expected to be very small, and 7) the infectivity of investigated vaccine and booster against the variant causing COVID-19 infection, and 8) an understanding or confirmation of SARS-CoV-2 presence during the post-infection recovery.

To manage these challenges, the best R&D suggestions were so far focused on the re-engineering of vaccines, virus-free indoor air, antimicrobial coating on personal protective equipment, and development of nanostructures-based biosensors (optical, electrical, and magnetic) for the quantitative detection of SARS-CoV-2 at low levels. In this direction, special attention was paid to developing efficient SARS-CoV-2 sensing systems using (1) appropriate bio-recognizing agents such as antibodies concerning S-1 (major portion of total virus surface), genomic segments (nucleocapsid), and edited genes, i.e., CRISPR-Cas system, for selective sensing of SARS-CoV-2 and (2) nanostructures such as graphene, nano-plasmonic platforms, metal nanoparticle functionalized systems, and polymers to achieve high sensitivity and low detection limit.

An example is a terahertz plasmonic meta-sensor for SARS-CoV-2 detection (2.4 fM) using specific antibodies designed specific to the S-1 protein of the virus and immobilized onto the AuNPs functionalized magneto-plasmonic nanostructure. As a direct read-out, a graphene-modified capacitance interdigitated immunosensing chip was developed to detect SARS-CoV-2 at 1 fg/mL within 3 s. A CRISPR-Cas 12/gRNA-based assay was developed to identify specific RNA of SARS-CoV-2 (two copies per sample in 50 min) to discriminate symptomatic and pre-symptomatic conditions. A minimally instrumented SHERLOCK (miSHERLOCK)-supported CRISPR-based POC system of high sensitivity (2- to 20-fold) was developed for the multi-plex sensing of SARS-CoV-2 mutations (B.1.1.7, B.1.351, and P1) in 55 min. Recently, as a non-invasive sensing approach suitable for POCT, a porous microneedle-based immunochromatographic assay has been developed using IgG and IgM antibodies for the detection of SARS-CoV-2 (3–7 ng/mL within 3 min) selectively in dermal interstitial fluid.

The aforementioned biosensing prototypes are efficient in detecting SARS-CoV-2 selectively and fulfill the criteria for POC sensing due to desired miniaturization and easy operation. However, the validation of these technologies using a large clinical sample number is urgently required before projecting them for translational and clinical applications,^4^ Therefore, to manage COVID-19 in a personalized manner, there is an unmet need to explore high-performance nano-enabled biosensing components for developing affordable POC systems, and validation using real samples, and concerning SARS-CoV-2 variants. However, there is another urgent need to correlate the SARS-CoV-2 sensing outcomes with other aspects of health consequences because SARS-CoV-2 variants causing the infection are an emerging challenge, and they affect body function for a long time, as discussed in the following section.

## Challenges and prospects of biosensing to manage long COVID

SARS-CoV-2 mutations based on COVID-19 infection are studied mainly considering acute effects including multiple organ damage, mainly lungs, liver, kidney, digestive tract, heart, and a new concern of the brain. Recently, it has also been suggested to understand the long COVID via the understanding of chronic effects of SARS-CoV-2 infection for a long time, mainly more than 4 weeks. This is important in the case of mutated SARS-CoV-2, which can invade the vaccine and may cause re-occurrence of COVID-19 even after full vaccination and booster(s). To handle such scenarios, regular diagnostics of an individual who has been infected with SARS-CoV-2 are pivotal. In this direction, digital diagnostics will be the primary requirement to have personal health profiling that is supported by National COVID Cohort Collaborative’s (N3C) approach based on machine-learning-based modeling such as XGBoost to support established diagnostic systems to understand long COVID, i.e., chronic SARS-CoV-2 infections.^5^

**Figure 1:**
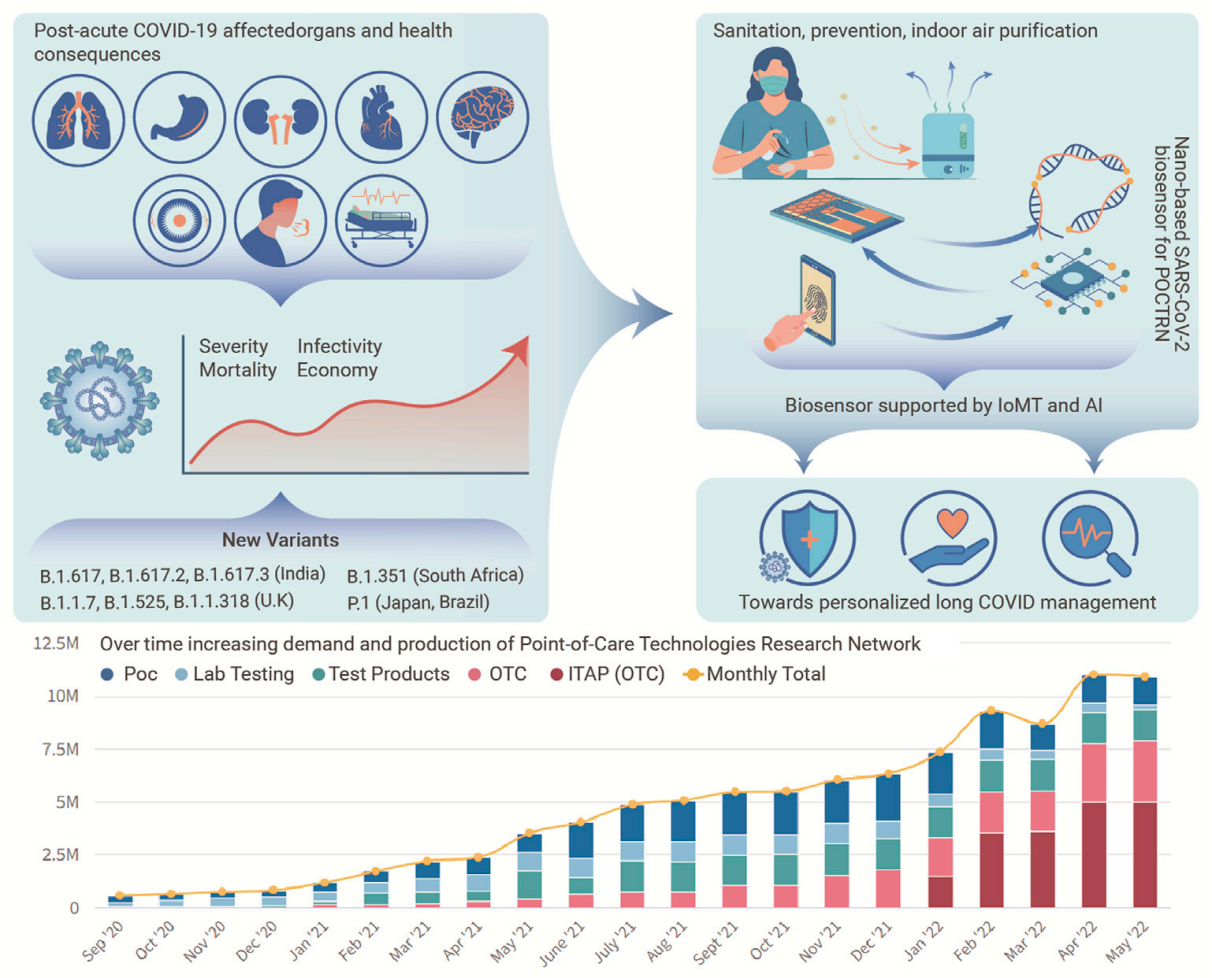
Schematic presentation of the status of the COVID-19 pandemic and recommendations on the development of sensitive smart SARS-CoV-2 sensing platforms using a combinatory approach on biosensors supported with internet-of-medical-things (IoMT) and artificial intelligence (AI). Point-of-care technologies research network (POCTRN); statistical presentation of tests produced per day by manufacturer including lab products, lab-based technologies, over the counter (OTC), and POC at home. Figure reprinted from the NIH dashboard https://www.nibib.nih.gov/covid-19/radx-tech-program/radx-tech-dashboard.

Such approaches are diverting the presenting research toward COVID-19 management in a personalized manner. Not only the management, but intelligent management of mutated SARS-CoV-2 infection, was more under consideration via integrating the developed SARS-CoV-2 biosensing prototype with internet-of-medical-things (IoMT) for device miniaturization and its further interfacing with artificial intelligence (AI)-assisted predictive analysis (Figure 1). Finally, the IoMT-based POCT of SARS-CoV-2 and analysis of generated bioinformatics using AI will give an idea about diseases condition and possibilities of severe health consequences due to the virus, mutation, and patient’s pre-infection medical condition.

The proposed combinational approach in this editorial is challenging because it requires a multidisciplinary R&D strategy that could be the focus of national agencies such as the RAD_x_Tech/ATP program of NIH to promote such technologies and advertise (1) technological development according to the need and timelines, (2) promoting POC systems suitable to use at home as well and with the capability of scaling-up to fulfill the global demand, (3) developing a collaborative network to increase partnership among various laboratories of the same or other institutes/companies, (4) promoting awareness for biosensor validation, (5) developing more studies focused on long COVID, and (6) connecting people, patients, or participants for enough sampling and databases.

## Conclusions and viewpoint

This editorial supports the objectives and outcomes of the RAD_x_Tech/ATP program of NIH focused on SSS development for SARS-CoV-2 biosensing assisted by IoMT and AI to perform sensing for a long time, as well as correlation of observed sensing informatics with the patient health conditions. These tasks could be achieved affordably and acceptably via performing a collaborative and well-partnered R&D according to the aims and scope of the RAD_x_Tech/ATP program, but it should be done globally and not only at the level of the United States.
